# NDM-Producing *Klebsiella pneumoniae* Bloodstream Infection Increases Mortality and Impairs Hematopoietic Recovery in Acute Leukemia

**DOI:** 10.3390/jcm15166259

**Published:** 2026-08-13

**Authors:** Krzysztof Gawronski, Nadia Hussein, Agnieszka Woźniak-Kosek, Piotr Rzepecki, Aneta Guzek

**Affiliations:** 1 Department of Internal Medicine and Hematology, Military Institute of Medicine—National Research Institute, Szaserow Str. 128, 04-141 Warsaw, Poland; 2 Department of Laboratory Diagnostics, Section of Microbiology, Military Institute of Medicine—National Research Institute, 04-141 Warsaw, Poland

**Keywords:** *Klebsiella pneumoniae*, New Delhi metallo-β-lactamase (NDM), bloodstream infection, acute leukemia, mortality, neutrophil recovery, hematopoietic recovery

## Abstract

**Background:** Bloodstream infections (BSIs) remain among the most serious complications of intensive chemotherapy for acute leukemia. Infections caused by New Delhi metallo-β-lactamase (NDM)-producing *Klebsiella pneumoniae* are associated with limited therapeutic options and poor prognosis; however, their impact on hematopoietic recovery has not been comprehensively evaluated. We investigated the association of NDM-producing *K. pneumoniae* BSI with mortality and hematopoietic recovery in patients with acute leukemia. **Methods**: This retrospective single-center cohort study included 255 microbiologically documented BSI episodes occurring in 201 adult patients with acute myeloid leukemia or acute lymphoblastic leukemia between January 2020 and December 2025. Episodes in the NDM group (*n* = 103) were compared with BSIs caused by other bacterial or fungal pathogens (*n* = 152). The primary endpoint was all-cause mortality. Secondary endpoints included the proportion achieving neutrophil and reticulocyte recovery, the cumulative incidence of neutrophil recovery, time to hematopoietic recovery among patients who survived and achieved recovery, and septic shock. Neutrophil recovery was additionally evaluated using a competing-risk approach, with death before recovery treated as a competing event. Independent predictors of mortality were identified using multivariable logistic regression. **Results**: Patients in the NDM group experienced significantly higher mortality than those in the comparator group (36.9% vs. 12.5%; OR 4.09, 95% CI 2.19–7.65; *p* < 0.001). Neutrophil recovery was achieved less frequently in the NDM group (63.1% vs. 87.5%; OR 0.24, 95% CI 0.13–0.46; *p* < 0.001). Among patients who survived and achieved hematopoietic recovery, the median time to neutrophil recovery was shorter in the NDM group (9 vs. 15 days; *p* < 0.001), an observation interpreted in the context of survivorship bias rather than accelerated marrow regeneration. The competing-risk analysis similarly demonstrated a lower cumulative probability of neutrophil recovery in the NDM group when death before recovery was considered as a competing event. Multivariable analysis identified NDM-producing *K. pneumoniae* BSI (adjusted OR 6.03, 95% CI 3.06–11.87), increasing age (adjusted OR 1.04 per year, 95% CI 1.01–1.06), and septic shock (adjusted OR 3.84, 95% CI 1.94–7.60) as independent predictors of mortality. **Conclusions:** NDM-producing *K. pneumoniae* bloodstream infection was independently associated with substantially increased mortality and a markedly reduced probability of hematopoietic recovery in patients with acute leukemia. The apparently shorter recovery time among patients who survived and achieved recovery should be interpreted in the context of selective early mortality rather than faster marrow regeneration. Assessment of severe bloodstream infections in acute leukemia should therefore integrate survival and hematopoietic recovery and account for death before recovery as a competing event.

## 1. Introduction

Patients with acute leukemia are highly susceptible to infectious complications due to disease-related immune dysfunction, intensive cytotoxic chemotherapy, prolonged neutropenia, disruption of mucosal barriers, and frequent exposure to broad-spectrum antimicrobial agents. Bloodstream infections (BSIs) remain one of the leading causes of morbidity and mortality in this population and frequently complicate periods of profound chemotherapy-induced aplasia [1,2].

Over the past two decades, the epidemiology of bloodstream infections in hematology departments has changed substantially as a consequence of the global emergence and dissemination of multidrug-resistant Gram-negative bacteria. Among these pathogens, carbapenem-resistant Enterobacterales (CRE) have become a major therapeutic and epidemiological challenge. Infections caused by CRE are associated with limited treatment options, delayed administration of effective antimicrobial therapy, prolonged hospitalization, increased healthcare costs, and poor clinical outcomes, including high mortality rates [3,4,5].

One of the most clinically relevant mechanisms of carbapenem resistance is the production of carbapenem-hydrolyzing enzymes, particularly New Delhi metallo-β-lactamases (NDMs). NDM enzymes were first described in 2008 in a carbapenem-resistant *Klebsiella pneumoniae* isolate recovered from a Swedish patient who had previously received medical care in India. Since then, NDM-producing Enterobacterales have disseminated worldwide and have become endemic in many geographical regions, including Eastern Europe and parts of Asia [6,7].

Among patients with hematological malignancies, *Klebsiella pneumoniae* is one of the most clinically important CRE pathogens. Previous studies have demonstrated that bloodstream infections caused by carbapenem-resistant *K. pneumoniae* (CRKP) are associated with mortality rates ranging from approximately 30% to more than 70%, depending on patient characteristics, severity of neutropenia, timing of active antimicrobial therapy, and the presence of septic shock or organ dysfunction [8,9,10,11]. Delayed initiation of appropriate antimicrobial treatment, intensive care unit admission, prolonged neutropenia, and severe sepsis have consistently been identified as major predictors of adverse outcomes [8,9,10].

Intestinal colonization with CRE has also emerged as a critical determinant of subsequent invasive infection. In patients with hematological malignancies, gastrointestinal colonization frequently precedes bloodstream infection and is associated with a markedly increased risk of CRE bacteremia and infection-related mortality. Consequently, active surveillance cultures and early identification of colonized patients have become integral components of infection prevention and control programs in many hematology centers [12,13,14]. Importantly, Castañón et al. demonstrated in patients with acute myeloid leukemia that a comprehensive strategy incorporating systematic surveillance cultures, infection-control measures, and targeted antimicrobial management was associated with a reduction in multidrug-resistant Gram-negative infections and infectious mortality [15].

Although the impact of CRE bloodstream infections on mortality has been extensively investigated, considerably less attention has been paid to hematopoietic recovery following infection. Restoration of neutrophil production is a fundamental prerequisite for effective infection control in patients with acute leukemia. Failure to achieve neutrophil recovery may contribute to persistent infection, prolonged exposure to antimicrobial therapy, multiple organ dysfunction, interruption or delay of antileukemic treatment, and death. Nevertheless, most previous studies have focused primarily on microbiological characteristics, antimicrobial management, and mortality, whereas dynamic parameters of hematopoietic regeneration have rarely been evaluated as clinically meaningful outcomes [8,15].

The present study was designed to evaluate the clinical impact of bloodstream infections caused by NDM-producing *Klebsiella pneumoniae* in adult patients with acute leukemia. Specifically, we compared mortality, hematopoietic recovery, and the occurrence of severe infectious complications between NDM-producing *K. pneumoniae* bloodstream infections and bloodstream infections caused by other bacterial or fungal pathogens. Chemotherapy treatment phase at the time of BSI was additionally recorded to provide clinical context for the observed infectious episodes.

## 2. Materials and Methods

### 2.1. Study Design and Patient Population

This retrospective cohort study was conducted at the Department of Internal Medicine and Hematology, Military Institute of Medicine—National Research Institute (WIM-PIB), Warsaw, Poland. Adult patients diagnosed with acute myeloid leukemia (AML) or acute lymphoblastic leukemia (ALL) who developed microbiologically confirmed bloodstream infection (BSI) between January 2020 and December 2025 were eligible for inclusion.

The study was performed at the episode level because individual patients could experience more than one microbiologically documented bloodstream infection during the observation period. Overall, 255 microbiologically confirmed BSI episodes were analyzed, including 103 episodes caused by NDM-producing *Klebsiella pneumoniae* and 152 episodes caused by other bacterial or fungal pathogens. Episodes occurring during induction chemotherapy, consolidation chemotherapy, second induction (salvage therapy), or chemotherapy-induced aplasia were included.

### 2.2. Microbiological Procedures

Routine microbiological surveillance for multidrug-resistant organisms was performed throughout hospitalization as part of the institutional infection prevention program. Surveillance cultures were obtained on admission and subsequently according to local clinical practice.

Nasal swabs were used for detection of methicillin-resistant *Staphylococcus aureus* (MRSA), whereas rectal swabs were collected to identify colonization with vancomycin-resistant enterococci (VRE), extended-spectrum β-lactamase (ESBL)-producing Enterobacterales, and carbapenemase-producing Enterobacterales, including isolates producing New Delhi metallo-β-lactamase (NDM) and OXA-48 carbapenemases.

Specimens were cultured on selective chromogenic media (chromID MRSA, chromID VRE, chromID ESBL, chromID CARBA SMART, and chromID OXA-48; bioMérieux, Marcy-l’Étoile, France). Microorganisms recovered from surveillance cultures were identified by matrix-assisted laser desorption/ionization time-of-flight mass spectrometry (MALDI-TOF MS; VITEK^®^ MS, bioMérieux, Marcy-l’Étoile, France).

### 2.3. Bloodstream Infection Diagnosis

Blood cultures were obtained whenever bloodstream infection was clinically suspected on the basis of fever, signs of sepsis, or other manifestations suggestive of systemic infection. For each suspected episode, at least two blood culture sets, each consisting of one aerobic and one anaerobic bottle, were collected before initiation or modification of antimicrobial therapy whenever feasible.

Cultures were processed using the BacT/ALERT^®^ VIRTUO^®^ automated blood culture system (bioMérieux, Marcy-l’Étoile, France). Positive bottles underwent Gram staining followed by rapid molecular identification with the BIOFIRE^®^ Blood Culture Identification 2 (BCID2) Panel (BioFire Diagnostics, LLC, Salt Lake City, UT, USA). Definitive species identification was subsequently confirmed by MALDI-TOF MS using the VITEK^®^ MS system (bioMérieux, Marcy-l’Étoile, France). 

When identical microorganisms were repeatedly isolated during a single infectious episode, only the first isolate was included in the statistical analyses. Blood culture contaminants were excluded according to predefined microbiological and clinical criteria.

### 2.4. Antimicrobial Susceptibility Testing

Antimicrobial susceptibility testing was performed using the automated VITEK^®^ 2 system (bioMérieux). Results were interpreted according to the current European Committee on Antimicrobial Susceptibility Testing (EUCAST) clinical breakpoints valid at the time of isolate identification.

ESBL production was confirmed using the double-disk synergy test. Carbapenemase production was verified by the RESIST-5 immunochromatographic assay and molecular testing when clinically indicated. Methicillin resistance in *S. aureus* was determined using cefoxitin susceptibility testing, whereas vancomycin resistance in enterococci was confirmed by gradient diffusion (E-test). Colistin susceptibility was assessed exclusively by reference broth microdilution in accordance with EUCAST recommendations.

### 2.5. Clinical Variables and Outcome Definitions

Clinical information was extracted retrospectively from electronic medical records, microbiological databases, and institutional infection surveillance records. Collected variables included age, sex, leukemia subtype, chemotherapy treatment phase, microbiological findings, previous colonization with multidrug-resistant organisms, septic shock, multiple organ dysfunction syndrome (MODS), hematopoietic recovery, and survival.

The primary study outcome was all-cause mortality. Secondary outcomes included the proportion achieving neutrophil and reticulocyte recovery, the cumulative incidence of neutrophil recovery, time to hematopoietic recovery among patients who survived and achieved recovery, and the occurrence of septic shock.

Neutrophil recovery was defined as the first documented absolute neutrophil count (ANC) exceeding 0.5 × 10^9^/L after bloodstream infection. Reticulocyte recovery was defined as achievement of a reticulocyte production index (RPI) > 1.0. Patients who died before meeting these criteria were classified as having failed hematopoietic recovery.

Hematopoietic recovery was evaluated using complementary measures. The proportion achieving recovery was defined as the number of evaluable episodes in which the predefined recovery criterion was achieved divided by the total number of evaluable episodes in the corresponding group; patients who died before recovery were classified as not having achieved recovery. In addition, the time-dependent probability of neutrophil recovery was estimated using the cumulative incidence function, with death before neutrophil recovery treated as a competing event. Time to neutrophil recovery among patients who survived and achieved recovery was analyzed separately and was not used to estimate the overall probability of recovery.

The chemotherapy treatment phase was recorded at the time of each BSI episode and classified as induction, consolidation, or second induction (salvage). Because the study database was constructed at the BSI-episode level, chemotherapy cycles during which no BSI occurred were not systematically captured. Consequently, treatment-phase data were used to describe the distribution of BSI episodes across chemotherapy phases and not to estimate phase-specific BSI incidence per administered chemotherapy cycle.

### 2.6. Statistical Analysis

Continuous variables were summarized using medians and interquartile ranges (IQRs), whereas categorical variables were presented as counts and percentages. Between-group comparisons were performed using the Mann–Whitney U test for continuous variables and the chi-square test or Fisher’s exact test, as appropriate, for categorical variables.

To reduce survivorship bias, hematopoietic recovery was evaluated using two complementary approaches. First, the proportion achieving recovery was evaluated in the entire study cohort. Second, among patients who survived and achieved hematopoietic recovery, the interval from bloodstream infection to neutrophil recovery and to reticulocyte recovery was analyzed separately. This strategy enabled discrimination between delayed marrow regeneration and failure to recover due to early mortality.

Time-to-event outcomes were analyzed using survival methods. Overall survival was estimated using the Kaplan–Meier method and compared between groups using the log-rank test. The cumulative incidence of neutrophil recovery was estimated in a competing-risk framework, with neutrophil recovery considered the event of interest and death before neutrophil recovery treated as a competing event; cumulative incidence functions were compared using Gray’s test. Among patients who survived and achieved neutrophil recovery, time to neutrophil recovery was analyzed using Kaplan–Meier methods and compared between groups using the log-rank test.

Differences in hematopoietic recovery according to NDM infection status were evaluated using categorical comparisons and expressed as odds ratios (ORs) with 95% confidence intervals (CIs). The primary comparison included the overall NDM group and the comparator group. Additional subgroup analyses were performed separately for patients with confirmed NDM-producing *Klebsiella pneumoniae* bloodstream infection and those classified as NDM carriage only. These analyses were based on the final clinically verified hematopoietic recovery status.

Independent predictors of all-cause mortality were identified using multivariable logistic regression. Variables entered into the multivariable model were selected according to clinical relevance and the results of univariable analyses. Adjusted odds ratios (aORs) with 95% confidence intervals are reported.

All statistical tests were two-sided, and a *p* value < 0.05 was considered statistically significant. Statistical analyses were performed using Statistica version 13 (TIBCO Software Inc., Palo Alto, CA, USA).

### 2.7. Sensitivity Analyses

Sensitivity analyses were performed to assess the robustness of the primary findings. To distinguish the effect of active NDM-producing Klebsiella pneumoniae bloodstream infection from that of NDM carriage alone, analyses of hematopoietic recovery were repeated after restricting the NDM group to episodes with confirmed NDM-producing *K. pneumoniae* bloodstream infection. Patients classified as NDM carriage only were also evaluated separately.

Hematopoietic recovery was additionally assessed using complementary approaches that accounted for early mortality. The proportion achieving recovery was evaluated in the entire cohort, while time to neutrophil and reticulocyte recovery was assessed among patients who survived and achieved hematopoietic recovery. In addition, cumulative incidence of neutrophil recovery was evaluated with death before recovery treated as a competing event. These analyses were considered supportive and were used to assess the robustness and consistency of the primary findings.

## 3. Results

### 3.1. Study Population

A total of 255 microbiologically confirmed bloodstream infection episodes occurring in 201 adult patients with acute leukemia were included in the analysis. Among these episodes, 103 (40.4%) were caused by NDM-producing *Klebsiella pneumoniae*, whereas 152 (59.6%) were caused by other bacterial or fungal pathogens.

Baseline demographic and clinical characteristics are summarized in Table 1. Patients in the NDM group were slightly younger than those in the comparator group (median age 34 vs. 38 years, *p* = 0.034), whereas sex distribution and leukemia subtype were similar between groups. The distribution of BSI episodes according to chemotherapy treatment phase is presented descriptively in Table 1. Among NDM-producing *K. pneumoniae* BSI episodes, 58 (56.3%) occurred during induction, 27 (26.2%) during consolidation, and 18 (17.5%) during second induction. Among BSI episodes caused by other microorganisms, the corresponding numbers were 84 (55.3%), 36 (23.7%), and 31 (20.4%), respectively. These proportions describe the distribution of BSI episodes across treatment phases and do not represent phase-specific BSI incidence per administered chemotherapy cycle. As expected, previous colonization with NDM-producing *K. pneumoniae* and colonization with multidrug-resistant Gram-negative organisms was significantly more frequent among patients who subsequently developed NDM bloodstream infection.

### 3.2. Microbiological Characteristics

The microbiological spectrum of bloodstream infections in the comparator group is presented in Table 2.

Among Gram-negative pathogens, *Escherichia coli* was the most frequently isolated organism, followed by non-NDM *Klebsiella pneumoniae*. Gram-positive bloodstream infections were predominantly caused by *Staphylococcus epidermidis* and *Enterococcus faecium*, including vancomycin-resistant isolates. Fungal bloodstream infections were uncommon and accounted for less than 2% of all comparator episodes.

Overall, the comparator cohort represented a broad spectrum of clinically relevant bloodstream pathogens encountered in patients with acute leukemia.

### 3.3. Clinical Outcomes

Clinical outcomes according to bloodstream infection etiology are summarized in Table 3. Patients with NDM-producing *Klebsiella pneumoniae* bloodstream infection experienced significantly worse outcomes than those infected with other microorganisms. All-cause mortality was nearly threefold higher in the NDM group (36.9% vs. 12.5%; OR 4.09, 95% CI 2.19–7.65; *p* < 0.001).

**Figure 1:**
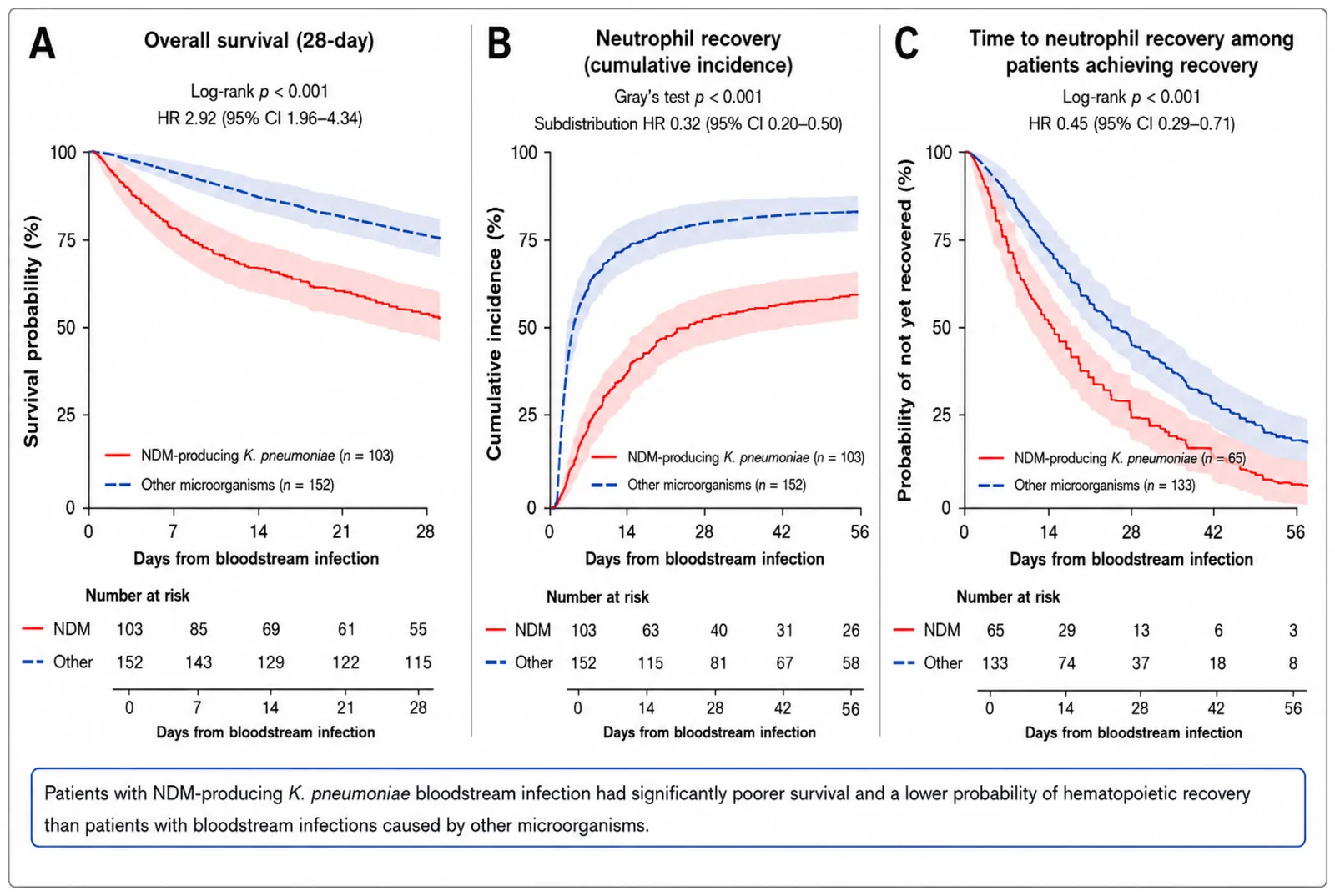
Clinical outcomes according to bloodstream infection etiology. (**A**) Overall 28-day survival. (**B**) Cumulative incidence of neutrophil recovery accounting for death as a competing event. (**C**) Time to neutrophil recovery among patients who survived and achieved neutrophil recovery. Shaded areas represent 95% confidence intervals. Abbreviations: CI, confidence interval; HR, hazard ratio; NDM, New Delhi metallo-β-lactamase.

The proportion achieving hematopoietic recovery was also significantly lower following NDM bloodstream infection. Neutrophil recovery was achieved in only 63.1% of patients with NDM-producing *K. pneumoniae* bloodstream infection compared with 87.5% of patients in the comparator group (OR 0.24, 95% CI 0.13–0.46; *p* < 0.001). Consistent with these findings, Figure 1A demonstrates significantly reduced 28-day overall survival among patients with NDM-producing *K. pneumoniae* bloodstream infection compared with those infected by other microorganisms.

Among patients who survived and achieved hematopoietic recovery, the interval from bloodstream infection to neutrophil regeneration was significantly shorter in the NDM group (median 9 vs. 15 days; *p* < 0.001). Figure 1B presents the cumulative incidence of hematopoietic recovery while accounting for death as a competing event, demonstrating that the lower recovery probability observed in the NDM group was primarily driven by excess early mortality rather than delayed bone marrow regeneration among surviving patients.

A similar pattern was observed for erythropoietic recovery. Achievement of a reticulocyte production index (RPI) > 1.0 before death was significantly less frequent in the NDM group, whereas surviving patients reached this endpoint earlier than those in the comparator group (median 12 vs. 19 days; *p* < 0.001). These findings further support the conclusion that the apparent shortening of recovery time reflects selective survival rather than accelerated hematopoietic recovery.

Among patients who survived and achieved neutrophil recovery, Figure 1C demonstrates a shorter time to neutrophil recovery in the NDM group compared with the comparator group (log-rank *p* < 0.001). This finding should be interpreted in the context of the substantially higher early mortality in the NDM group and supports the interpretation that the lower overall probability of hematopoietic recovery was primarily related to death before recovery rather than prolonged neutrophil regeneration among survivors.

Taken together, the findings presented in Table 3 and Figure 1 demonstrate that NDM-producing *Klebsiella pneumoniae* bloodstream infection adversely affects both survival and hematopoietic recovery. The reduced probability of marrow recovery observed in the NDM group primarily reflects excess early mortality rather than delayed hematopoietic regeneration among patients who survived the acute infectious episode.

### 3.4. Hematopoietic Recovery According to NDM Infection Status

Hematopoietic recovery according to NDM infection status is summarized in Table 4. In the overall NDM group, hematopoietic recovery was achieved in 65 of 103 episodes (63.1%), compared with 133 of 152 episodes (87.5%) in the comparator group. Accordingly, failure to achieve hematopoietic recovery was significantly more frequent in the NDM group (38/103 [36.9%] vs. 19/152 [12.5%]; OR 4.09, 95% CI 2.19–7.65; *p* < 0.001).

When the analysis was restricted to episodes with confirmed NDM-producing *Klebsiella pneumoniae* bloodstream infection, excluding episodes classified as NDM carriage only, hematopoietic recovery was achieved in 43 of 78 episodes (55.1%), whereas 35 of 78 episodes (44.9%) failed to achieve recovery. Compared with bloodstream infections caused by other microorganisms, confirmed NDM-producing *K. pneumoniae* BSI was associated with substantially higher odds of failure to achieve hematopoietic recovery (OR 5.70, 95% CI 2.96–10.98; *p* < 0.001).

In contrast, among the 25 episodes classified as NDM carriage only, hematopoietic recovery was achieved in 22 (88.0%), while 3 (12.0%) failed to achieve recovery. The odds of recovery failure in this subgroup did not differ from those observed in the comparator group (OR 0.95, 95% CI 0.26–3.50; *p* = 1.000). These findings indicate that the adverse association with hematopoietic recovery was concentrated among patients with confirmed NDM-producing *K. pneumoniae* bloodstream infection rather than those with NDM carriage alone.

### 3.5. Independent Predictors of Mortality

The results of the multivariable logistic regression analysis are summarized in Table 5. After adjustment for clinically relevant covariates, bloodstream infection caused by NDM-producing Klebsiella pneumoniae remained the strongest independent predictor of all-cause mortality (adjusted OR 6.03, 95% CI 3.06–11.87; *p* < 0.001). Increasing age was also independently associated with mortality (adjusted OR 1.04 per year, 95% CI 1.01–1.06; *p* = 0.003), as was the presence of septic shock (adjusted OR 3.84, 95% CI 1.94–7.60; *p* < 0.001).

The final multivariable model therefore identified NDM-producing *K. pneumoniae* bloodstream infection, increasing age, and septic shock as independent predictors of all-cause mortality. The adverse prognostic association of NDM-producing *K. pneumoniae* bloodstream infection remained significant after adjustment for the other covariates included in the final model.

Taken together, these findings demonstrate a strong association between NDM-producing *K. pneumoniae* bloodstream infection and adverse clinical outcomes in patients with acute leukemia, characterized by increased mortality and a lower probability of hematopoietic recovery.

## 4. Discussion

Bloodstream infections remain one of the leading causes of treatment-related mortality in patients with acute leukemia receiving intensive chemotherapy. The emergence of carbapenem-resistant Enterobacterales has further complicated infection management because these pathogens are associated with delayed administration of effective therapy, limited antimicrobial options, and poor clinical outcomes [3,4,5]. Among carbapenem-resistant organisms, NDM-producing *Klebsiella pneumoniae* represents a particularly important clinical challenge owing to the hydrolytic activity of metallo-β-lactamases, which renders many β-lactam/β-lactamase inhibitor combinations ineffective [6,7].

Previous studies have consistently demonstrated the adverse prognosis associated with carbapenem-resistant bloodstream infections in patients with hematological malignancies. Qian et al. identified carbapenem-resistant organism bloodstream infection as an important determinant of mortality and incorporated microbiological and clinical variables into a mortality prediction model for patients with hematological malignancies [16]. Trecarichi et al. demonstrated that carbapenem resistance itself was associated with significantly increased mortality among patients with hematological malignancies and bloodstream infection [17]. Subsequently, Satlin et al. confirmed that neutropenic patients with carbapenem-resistant Enterobacterales bacteremia constitute one of the highest-risk populations, emphasizing the importance of prompt initiation of active antimicrobial therapy [18]. More recently, Tang et al. and Ma et al. reported persistently high mortality despite improvements in microbiological diagnostics and supportive care [19,20]. Chen et al. subsequently confirmed these observations by integrating clinical and genomic analyses of carbapenem-resistant Enterobacterales bloodstream infections in patients with hematological malignancies [21].

The present study expands these observations in several important ways. First, bloodstream infection caused by NDM-producing *K. pneumoniae* was independently associated with increased all-cause mortality after adjustment for age and septic shock. Second, patients in the NDM group had a substantially lower probability of achieving hematopoietic recovery than those with bloodstream infections caused by other microorganisms. Third, the apparently shorter time to hematopoietic recovery among surviving patients with NDM infection should not be interpreted as accelerated marrow regeneration, but rather in the context of substantial early mortality and the resulting survivorship bias. Finally, the adverse association with hematopoietic recovery was concentrated among patients with confirmed NDM-producing *K. pneumoniae* bloodstream infection, whereas patients classified as NDM carriage only had recovery rates comparable to those observed in the non-NDM comparator group.

### 4.1. Hematopoietic Recovery: An Underrecognized Clinical Outcome

While increased mortality is the best-recognized consequence of carbapenem-resistant bloodstream infections, considerably less attention has been paid to their effect on subsequent hematopoietic recovery. Trecarichi et al. emphasized that successful management of severe infections in patients with hematological malignancies depends not only on microbiological eradication but also on restoration of host immune function [17]. Likewise, Satlin et al. highlighted the pivotal role of prolonged neutropenia in determining the outcome of carbapenem-resistant Enterobacterales bacteremia [18]. Nevertheless, recovery of hematopoiesis has rarely been evaluated as a predefined clinical endpoint in studies investigating multidrug-resistant bloodstream infections [19,21].

The present study addresses this gap by demonstrating that patients in the NDM group were significantly less likely to achieve neutrophil and reticulocyte recovery than patients infected with other microorganisms. From a clinical perspective, this observation is particularly relevant because hematopoietic recovery determines not only the ability to control the current infectious episode but also eligibility for subsequent chemotherapy cycles and, ultimately, continuation of curative-intent treatment [22].

An apparently paradoxical observation was that patients who survived and achieved recovery after NDM-associated episodes reached neutrophil and reticulocyte recovery earlier than patients in the comparator group. Importantly, this finding should not be interpreted as evidence of accelerated marrow regeneration. Patients who died before hematopoietic recovery were, by definition, unable to contribute subsequent recovery times. Consequently, analyses restricted to patients who survived and achieved recovery preferentially included individuals who had already survived the most critical phase of infection, thereby introducing survivorship bias.

This interpretation is supported by the competing-risk analysis. When death before neutrophil recovery was treated as a competing event, the cumulative probability of recovery remained substantially lower in the NDM group. Thus, the clinically relevant difference between groups was not simply the observed speed of marrow regeneration among survivors, but the markedly reduced probability of reaching hematopoietic recovery in the first place.

The distinction between active NDM-producing *K. pneumoniae* bloodstream infection and NDM carriage alone further strengthens this interpretation. Among episodes with confirmed NDM-producing *K. pneumoniae* BSI, hematopoietic recovery was achieved in 43 of 78 cases (55.1%), whereas 35 of 78 (44.9%) failed to achieve recovery. By contrast, 22 of 25 patients (88.0%) classified as NDM carriage only achieved recovery, a proportion comparable to that observed in the non-NDM comparator group. These findings suggest that the adverse association with hematopoietic recovery was concentrated among patients with active NDM-producing *K. pneumoniae* bloodstream infection rather than among those with NDM carriage alone.

By evaluating both the probability of hematopoietic recovery in the entire cohort and the time to recovery among survivors, our study provides a more comprehensive assessment of infection-related outcomes than either endpoint alone. Few previous investigations of carbapenem-resistant bloodstream infections in hematological patients have combined these complementary perspectives. Future clinical studies should therefore consider hematopoietic recovery together with mortality, as these outcomes reflect different but closely related dimensions of treatment success [23,24].

### 4.2. Influence of Chemotherapy Treatment Phase

The chemotherapy treatment phase is an important clinical consideration when interpreting hematopoietic recovery because the depth and duration of treatment-related myelosuppression differ across induction, consolidation, and salvage therapy. In the present cohort, BSI episodes occurred during all three treatment phases, and their distribution was broadly similar between the NDM and comparator groups.

However, the present database was constructed at the BSI-episode level and did not systematically capture chemotherapy cycles during which no bloodstream infection occurred. Consequently, the proportions of BSI episodes observed during induction, consolidation, and second induction should be interpreted descriptively and cannot be used to estimate phase-specific BSI incidence per administered chemotherapy cycle. This distinction is important because the number of BSI episodes observed within a given treatment phase is not equivalent to the risk of BSI among all chemotherapy cycles administered during that phase.

Accordingly, our findings do not permit definitive conclusions regarding whether the incidence of NDM-producing *K. pneumoniae* bloodstream infection differs independently between induction, consolidation, and second-induction therapy. Prospective studies capturing the complete denominator of administered chemotherapy cycles will be required to address this question reliably.

Nevertheless, microbiological characteristics and antimicrobial resistance remain major determinants of outcome in patients with hematological malignancies, alongside host- and treatment-related risk factors [20,21]. From a practical perspective, infection-prevention measures should therefore remain rigorous throughout the course of leukemia treatment. Continuous microbiological surveillance, rapid pathogen identification, strict infection-control measures, and early administration of active antimicrobial therapy remain essential components of care [23,25].

### 4.3. Clinical Implications

The findings of the present study have several important implications for the clinical management of patients with acute leukemia complicated by bloodstream infection. Previous evidence suggests that optimization of antimicrobial therapy, including appropriate treatment strategies for carbapenemase-producing *K. pneumoniae*, may improve outcomes in selected patients [23]. Current international recommendations emphasize prompt microbiological diagnosis, early administration of active antimicrobial therapy, and rigorous infection-control measures as cornerstones of the management of infections caused by multidrug-resistant Gram-negative bacteria [24].

Our findings suggest that NDM-producing *K. pneumoniae* bloodstream infection should be regarded not only as a marker of increased mortality risk but also as an indicator of a substantially reduced probability of successful clinical recovery. Patients who fail to restore neutrophil and reticulocyte production remain vulnerable to recurrent infections, prolonged hospitalization, treatment delays, and deterioration of their underlying hematological disease. Consequently, assessment of hematopoietic recovery provides clinically relevant information that complements conventional survival analyses and offers a broader measure of treatment success.

These observations also underline the importance of rapid identification of NDM-producing organisms. Previous studies have consistently demonstrated that delays in administration of effective antimicrobial therapy are associated with poorer outcomes in patients with carbapenem-resistant Enterobacterales bloodstream infections [17,18]. Early recognition of NDM production through rapid microbiological and molecular diagnostics, together with timely optimization of antimicrobial therapy, may therefore reduce the duration of uncontrolled infection and improve the probability of survival until marrow recovery occurs. Although our study was not designed to evaluate individual antimicrobial regimens, the observed association between NDM bloodstream infection and adverse clinical outcomes supports continued efforts to shorten the interval between microbiological diagnosis and initiation of appropriate treatment.

From a broader perspective, our results indicate that evaluation of infectious complications in hematological malignancies should extend beyond mortality alone. Future clinical studies should consider incorporating hematopoietic recovery as a predefined endpoint, particularly in populations with prolonged chemotherapy-induced neutropenia. Importantly, mortality before recovery should be accounted for explicitly, ideally through competing-risk approaches, rather than restricting analyses exclusively to patients who survive and subsequently recover. Adoption of standardized recovery endpoints could facilitate comparisons between studies and provide a more clinically meaningful assessment of therapeutic interventions [25,26].

### 4.4. Strengths and Limitations

The present study has several important strengths. First, it included a relatively large cohort of microbiologically documented episodes evaluated at a tertiary hematology center using standardized diagnostic procedures. Second, all microbiological investigations were performed according to uniform institutional protocols, minimizing variability in pathogen identification and antimicrobial susceptibility testing throughout the study period. Third, unlike most previous studies, we evaluated hematopoietic recovery as a predefined clinical endpoint in addition to mortality, thereby providing a broader assessment of the consequences of NDM-producing *K. pneumoniae* bloodstream infection.

An additional strength is the complementary assessment of hematopoietic recovery. We evaluated both the probability of recovery in the overall study population and recovery kinetics among patients who survived and achieved hematopoietic recovery. Furthermore, cumulative incidence of neutrophil recovery was evaluated with death before recovery treated as a competing event. This approach allowed the apparently shorter recovery time among survivors to be interpreted in the appropriate clinical context and reduced the risk of drawing misleading conclusions from survivor-only analyses.

The distinction between confirmed NDM-producing *K. pneumoniae* bloodstream infection and NDM carriage alone represents another clinically relevant aspect of the analysis. The markedly lower probability of hematopoietic recovery observed among patients with confirmed NDM BSI, but not among those classified as carriage only, provides additional evidence that active invasive infection is associated with the adverse clinical outcome observed in this cohort.

Several limitations should also be acknowledged. The retrospective design introduces the possibility of residual confounding despite adjustment for clinically relevant variables. Because the study was conducted at a single tertiary referral center with a high prevalence of multidrug-resistant Gram-negative pathogens, the generalizability of our findings to institutions with different epidemiological characteristics may be limited. In addition, detailed analyses of antimicrobial treatment strategies, including timing of active therapy and individual antimicrobial combinations, were beyond the scope of the present investigation and therefore could not be incorporated comprehensively into the multivariable models.

Importantly, the study database was constructed at the BSI-episode level and did not systematically capture chemotherapy cycles during which no BSI occurred. Consequently, although the treatment phase at the time of each BSI episode could be described, phase-specific BSI incidence per administered chemotherapy cycle could not be calculated. The present study therefore cannot determine whether the risk of BSI differs between induction, consolidation, and second-induction chemotherapy. This limitation should be considered when interpreting the treatment-phase data and highlights the need for prospective studies that capture the complete number of chemotherapy cycles administered.

Other factors potentially influencing hematopoietic recovery, including measurable residual disease status, baseline bone marrow reserve, and detailed chemotherapy dose intensity, were not available uniformly because of the retrospective design. Finally, although the overall cohort was relatively large, some subgroup analyses included limited numbers of patients, reducing the precision of individual estimates and warranting cautious interpretation.

### 4.5. Future Perspectives

The increasing global dissemination of NDM-producing Enterobacterales continues to pose a major challenge for hematology centers, particularly in regions where these organisms have become endemic [23]. Despite continuous advances in molecular diagnostics and the availability of newer antimicrobial agents, the prognosis of patients with NDM-producing bloodstream infections remains unsatisfactory [23,24]. Consequently, improving clinical outcomes will likely require an integrated strategy combining rapid microbiological diagnosis, effective infection prevention, antimicrobial stewardship, and optimization of supportive care rather than relying on antimicrobial therapy alone [27].

Recent studies further suggest that routine rectal surveillance cultures may identify patients at increased risk of subsequent carbapenem-resistant bloodstream infection and may contribute to improved clinical outcomes through earlier implementation of targeted preventive and therapeutic strategies [28]. The present finding that patients with NDM carriage alone had substantially better hematopoietic recovery than those with confirmed NDM bloodstream infection further emphasizes the clinical relevance of preventing progression from colonization to invasive disease.

Our findings also suggest that future investigations should expand the traditional evaluation of bloodstream infections beyond mortality. Clinical trials assessing novel antimicrobial strategies should consider incorporating standardized measures of hematopoietic recovery together with survival endpoints. Such an approach may provide a more complete assessment of treatment efficacy, particularly in patients with prolonged chemotherapy-induced neutropenia, in whom restoration of bone marrow function directly determines the ability to continue antileukemic therapy [22,26].

In addition, prospective multicenter studies are needed to validate our observations in larger and more heterogeneous patient populations. Future studies should prospectively record all administered chemotherapy cycles, thereby allowing phase-specific infection incidence to be calculated using appropriate denominators. They should also evaluate the influence of antimicrobial treatment strategies, timing of active therapy, duration of bacteremia, colonization status, and emerging resistance mechanisms on both survival and hematopoietic recovery. Better understanding of these interactions may facilitate development of individualized therapeutic strategies for patients at the highest risk of adverse outcomes [21,23,26,29].

## 5. Conclusions

Bloodstream infection caused by NDM-producing *Klebsiella pneumoniae* was independently associated with substantially increased all-cause mortality and a markedly reduced probability of hematopoietic recovery in patients with acute leukemia receiving intensive chemotherapy. The adverse clinical impact of NDM bloodstream infection extended beyond survival alone and was particularly evident among patients with confirmed NDM-producing *K. pneumoniae* bloodstream infection, whereas patients classified as NDM carriage only had substantially more favorable recovery outcomes.

The apparently shorter time to neutrophil recovery observed among patients who survived and achieved hematopoietic recovery should not be interpreted as accelerated marrow regeneration. Rather, this finding reflects survivorship bias resulting from the high proportion of patients who died before recovery could occur. Accordingly, evaluation of severe bloodstream infections in acute leukemia should integrate both survival and hematopoietic recovery and should account for death before recovery as a competing event.

The chemotherapy treatment phase remains an important clinical context for interpreting infectious outcomes and hematopoietic recovery. However, because chemotherapy cycles without bloodstream infection were not systematically captured in the present study, phase-specific BSI incidence per administered chemotherapy cycle could not be estimated. Future prospective studies should therefore record the complete number of chemotherapy cycles administered in order to determine more precisely whether infection risk and clinical outcomes differ across induction, consolidation, and second-induction therapy.

From a clinical perspective, these findings support rapid recognition of NDM-producing *K. pneumoniae* bloodstream infection, prompt initiation of effective antimicrobial therapy, and continued implementation of comprehensive infection-prevention strategies, including active surveillance, strict infection-control measures, rapid microbiological diagnostics, and antimicrobial stewardship. Prospective multicenter studies are warranted to validate these observations and to determine whether optimized preventive and therapeutic strategies can improve both survival and hematopoietic recovery in this particularly vulnerable patient population [30].

## Figures and Tables

**Table 1:** Baseline characteristics of bloodstream infection episodes according to etiology.

Variable	NDM-Producing *K. Pneumoniae* (*n* = 103)	Other Microorganisms (*n* = 152)	*p* Value
Demographic characteristics			
Age, years, median (IQR)	34 (27–53.5)	38 (29–57.5)	0.034
Male sex, *n* (%)	48 (46.6)	77 (50.7)	0.610
Underlying hematologic disease			
Acute myeloid leukemia (AML), *n* (%)	86 (83.5)	126 (82.9)	1.000
Acute lymphoblastic leukemia (ALL), *n* (%)	17 (16.5)	26 (17.1)	
Microbiological history before bloodstream infection			
Previous colonization with NDM-producing *K. pneumoniae*, *n* (%)	66 (64.1)	27 (17.8)	<0.001
Previous colonization with any multidrug-resistant Gram-negative organism, *n* (%)	70 (68.0)	73 (48.0)	0.002
Chemotherapy treatment phase ^†^			—
Induction chemotherapy, *n* (%)	58 (56.3)	84 (55.3)	
Consolidation chemotherapy, *n* (%)	27 (26.2)	36 (23.7)	
Second induction (salvage), *n* (%)	18 (17.5)	31 (20.4)	

Data are presented as median (interquartile range) or number (%). *p* values refer to comparisons between study groups. Abbreviations: AML, acute myeloid leukemia; ALL, acute lymphoblastic leukemia; IQR, interquartile range; NDM, New Delhi metallo-β-lactamase. ^†^ Chemotherapy treatment phase refers to the phase during which each BSI episode occurred. Percentages represent the distribution of BSI episodes across treatment phases within each microbiological group and should not be interpreted as phase-specific BSI incidence per administered chemotherapy cycle.

**Table 2:** Microbiological distribution of bloodstream infection episodes included in the comparator group.

Microorganism	Episodes, *n*	Percentage (%)
**Gram-negative bacteria**		
*Escherichia coli*	23	15.1
*Klebsiella pneumoniae* (non-NDM)	19	12.5
*Escherichia coli* ESBL	7	4.6
*Stenotrophomonas maltophilia*	5	3.3
*Pseudomonas aeruginosa*	2	1.3
*Enterobacter cloacae*	2	1.3
*Klebsiella pneumoniae* ESBL	2	1.3
*Enterobacter cloacae* ESBL	2	1.3
*Aeromonas sobria*	2	1.3
*Citrobacter koseri*	2	1.3
*Klebsiella oxytoca*	1	0.7
*Morganella morganii*	1	0.7
*Klebsiella variicola*	1	0.7
*Pseudomonas stutzeri*	1	0.7
*Acinetobacter baumannii*	1	0.7
**Gram-positive bacteria**		
*Staphylococcus epidermidis*	17	11.2
*Enterococcus faecium* VRE	13	8.6
*Enterococcus faecium*	11	7.2
*Staphylococcus aureus*	9	5.9
*Listeria monocytogenes*	5	3.3
*Enterococcus faecalis*	3	2.0
Other Gram-positive organisms	13	8.6
**Fungi**		
*Candida glabrata*	2	1.3
*Candida krusei*	1	0.7
**Total comparator group**	**152**	**100.0**

Data are presented as the number and percentage of bloodstream infection episodes in the comparator group. Bold indicates microbiological group headings and the total row. Abbreviations: ESBL, extended-spectrum beta-lactamase; NDM, New Delhi metallo-beta-lactamase; VRE, vancomycin-resistant enterococci.

**Table 3:** Clinical outcomes according to bloodstream infection etiology.

Outcome	NDM-Producing *K. Pneumoniae* BSI (*n* = 103)	Other Microorganisms (*n* = 152)	OR (95% CI)	*p* Value
**Severe clinical outcomes**				
All-cause mortality, *n* (%)	38/103 (36.9)	19/152 (12.5)	4.09 (2.19–7.65)	<0.001
Septic shock, *n* (%)	32/103 (31.1)	36/152 (23.7)	1.46 (0.83–2.57)	0.150
**Hematopoietic recovery**				
Neutrophil recovery achieved, *n* (%)	65/103 (63.1)	133/152 (87.5)	0.24 (0.13–0.46)	<0.001
Death before neutrophil recovery, *n* (%)	38/103 (36.9)	19/152 (12.5)	4.09 (2.19–7.65)	<0.001
Time to ANC recovery among recovered patients, days, median (IQR)	9.0 (8.0–12.0)	15.0 (10.0–23.0)	—	<0.001
RPI > 1.0 achieved, *n* (%)	65/103 (63.1)	133/152 (87.5)	0.24 (0.13–0.46)	<0.001
Time to RPI > 1.0 among recovered patients, days, median (IQR)	12.0 (10.0–14.0)	19.0 (14.0–27.0)	—	<0.001

Data are presented as n/N (%) or median (interquartile range). Odds ratios refer to the outcome shown in each row. Bold indicates outcome category headings. Abbreviations: ANC, absolute neutrophil count; BSI, bloodstream infection; CI, confidence interval; IQR, interquartile range; NDM, New Delhi metallo-beta-lactamase; OR, odds ratio; RPI, reticulocyte production index.

**Table 4:** Hematopoietic recovery according to NDM status and bloodstream infection etiology.

Study Group	Recovery Achieved, *n/N* (%)	Recovery Failed, *n/N* (%)	OR for Recovery Failure (95% CI)	*p* Value
Other microorganisms (reference)	133/152 (87.5)	19/152 (12.5)	Reference	—
NDM-producing *K. pneumoniae*, overall	65/103 (63.1)	38/103 (36.9)	4.09 (2.19–7.65)	<0.001
NDM-producing *K. pneumoniae* BSI only	43/78 (55.1)	35/78 (44.9)	5.70 (2.96–10.98)	<0.001
NDM carriage only	22/25 (88.0)	3/25 (12.0)	0.95 (0.26–3.50)	1.000

Data are presented as *n/N* (%). Recovery status reflects the final clinically verified classification. Odds ratios estimate the odds of failure to achieve hematopoietic recovery relative to the comparator group with bloodstream infection caused by other microorganisms. The NDM BSI-only and carriage-only rows are subgroup analyses of the overall NDM cohort and therefore should not be summed with the overall NDM row. Abbreviations: BSI, bloodstream infection; CI, confidence interval; NDM, New Delhi metallo-beta-lactamase; OR, odds ratio.

**Table 5:** Multivariable logistic regression analysis of factors independently associated with all-cause mortality.

Variable	Adjusted OR	95% CI	*p* Value
NDM-producing *Klebsiella pneumoniae* bloodstream infection	6.03	3.06–11.87	<0.001
Age, per 1-year increase	1.04	1.01–1.06	0.003
Septic shock	3.84	1.94–7.60	<0.001

The dependent variable was all-cause mortality. The final model included NDM-producing Klebsiella pneumoniae bloodstream infection, age, and septic shock. Adjusted odds ratios were estimated using multivariable logistic regression (*n* = 255). Abbreviations: CI, confidence interval; NDM, New Delhi metallo-beta-lactamase; OR, odds ratio.

## Data Availability

The original contributions presented in this study are included in the article. Further inquiries can be directed to the corresponding author.
